# Supplementary material for: Structural basis of leukotriene B4 receptor 1 activation
Source: Nat Commun. 2022 Mar 3;13:1156. doi: 10.1038/s41467-022-28820-9 (PMC8894450; doi:10.1038/s41467-022-28820-9)
Supplement: Supplementary file 1 — Supplementary Information [file 41467_2022_28820_MOESM1_ESM.pdf]

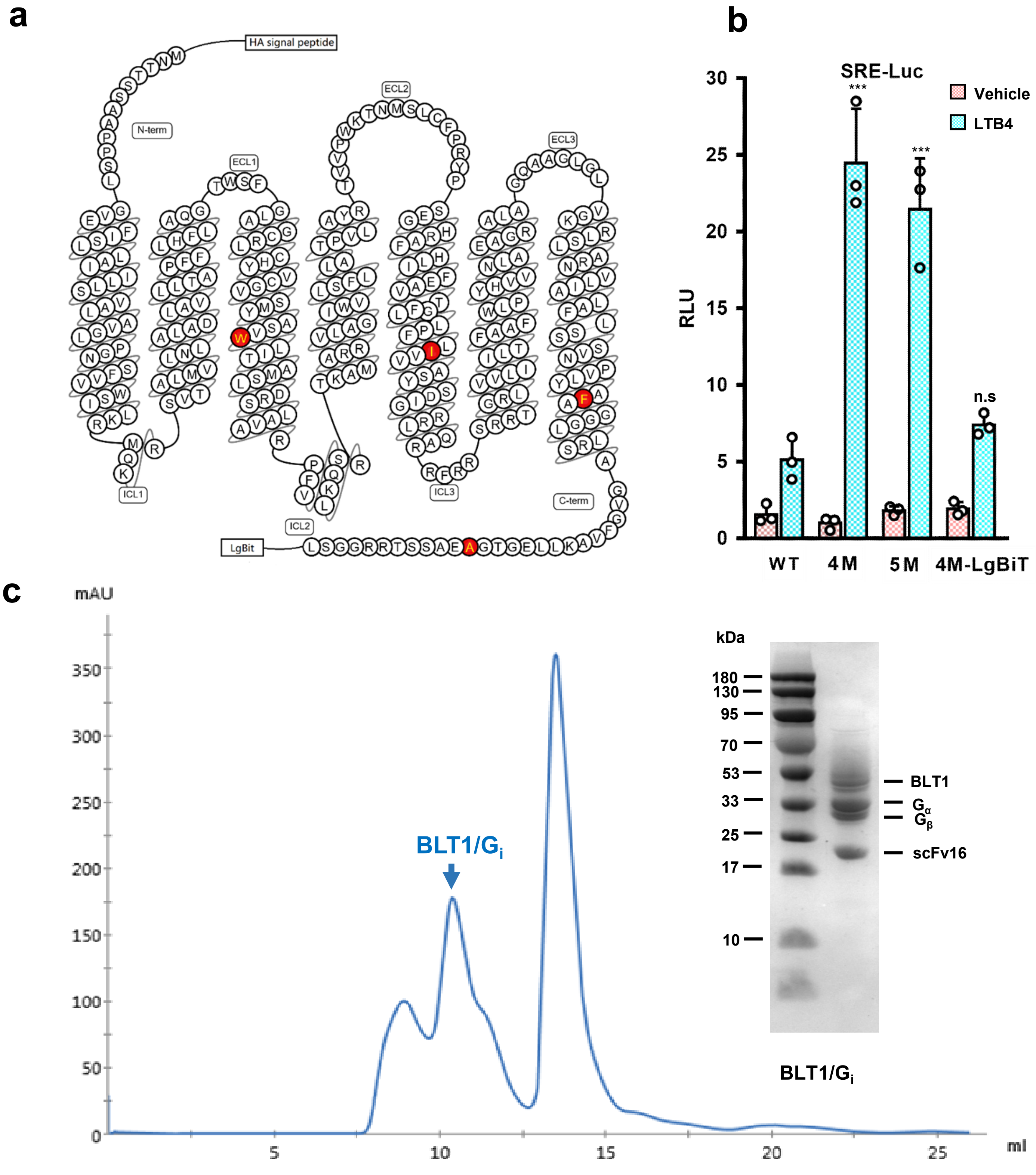

Supplementary Fig. 1. Construct and purification of the BLT1/Gi complex.
a, Snake-shape diagram of the BLT1 construct used in complex assembling, the snake-diagram of BLT1 was adopted from GPCRdb. The mutation was marked in red color. b, A SRE reporter assay to examine the effect of the 4 mutations and LgBiT fusion on the receptor activity. WT, wild-type; 4M, 4 mutations of L1063.41W, A1965.53I, C2877.55F and S310A; 5M, 4M plus S1163.51Y. 4M-LgBiT, a fusion of the LgBiT to the C-tail of the 4 mutations construct. LTB4, 300 nM; data are presented as mean values ± SD; n =3 independent samples; n.s. no significant; *, $p < 0.05$; **, $p < 0.01$; ***, $p < 0.001$. The exact p value for 4M, 5M, 4M-LgBit are: 0.0009, 0.001, 0.06, respectively. T-test, two tailed, sample equal variance. c, Size exclusion column profile of BLT1/Gi complex. Right panel, representative SDS PAGE gel of BLT1/Gi complex, the experiment has been repeated > 3 times.

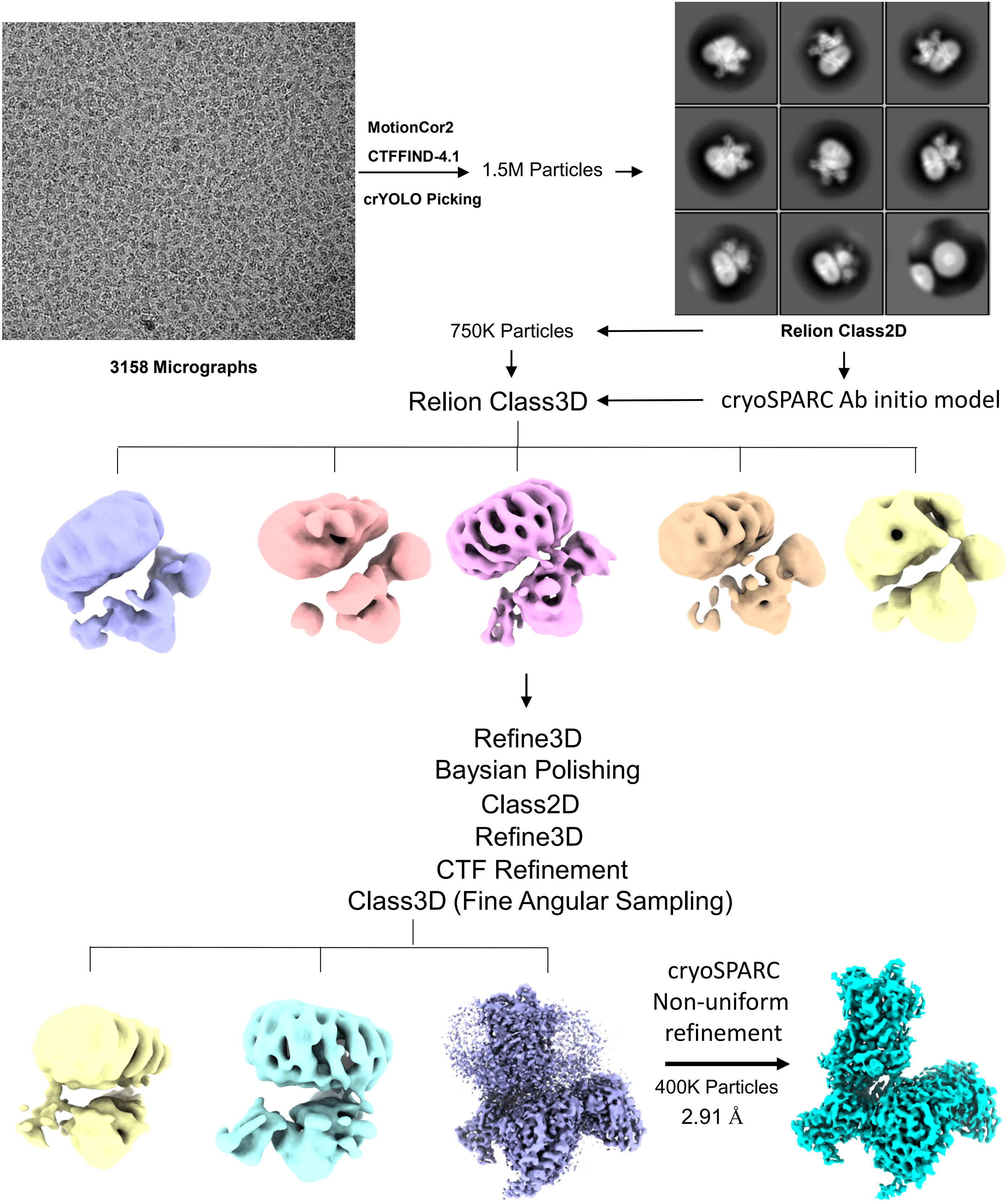

Supplementary Fig. 2. A flow-chart of the cryo-EM data process of the BLT1/Gi complex.

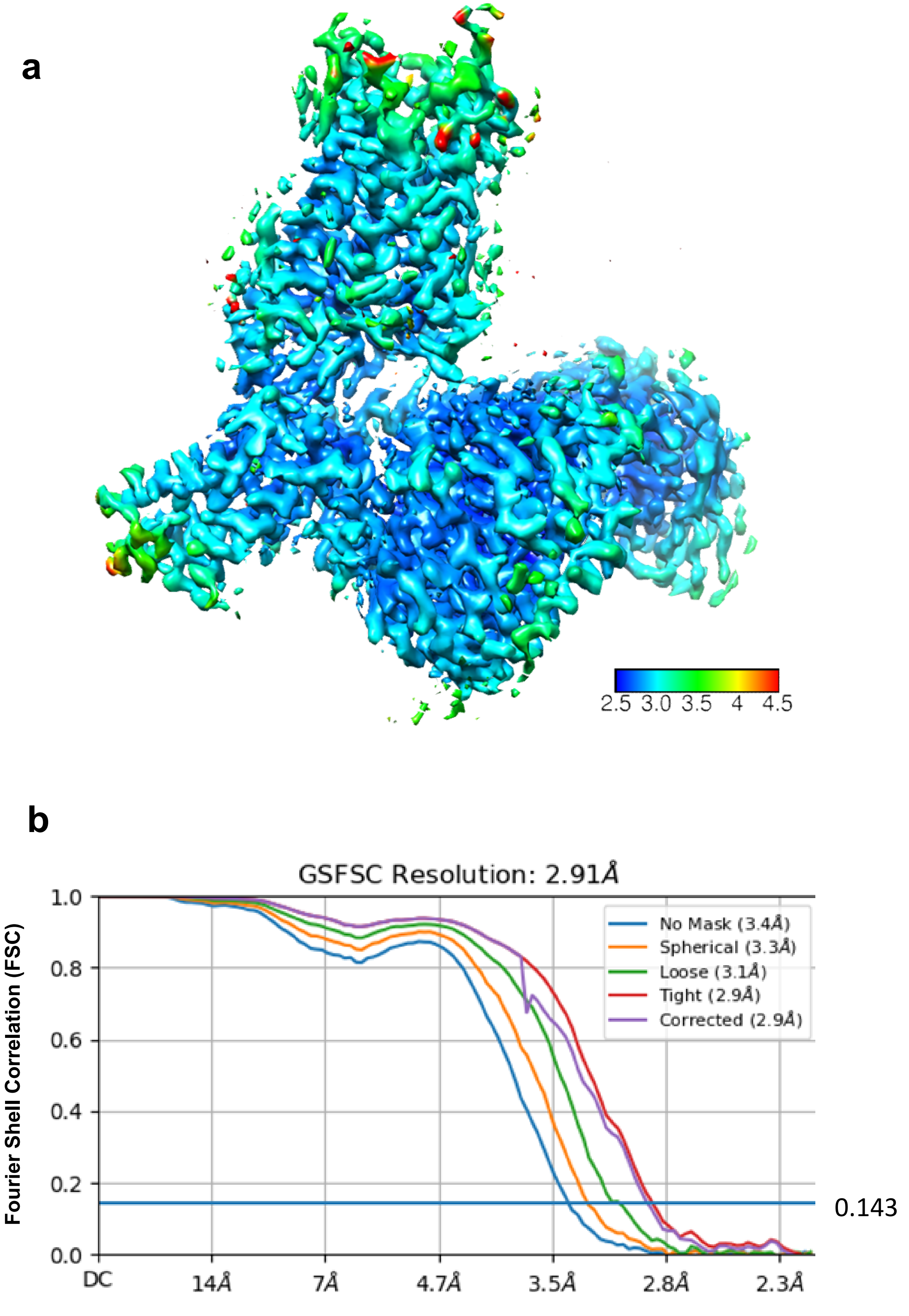

Supplementary Fig. 3. Resolution of the BLT1/Gi complex.
a, Local resolution analysis of the BLT1/Gi complex. b, FSC curve of the BLT1/Gi complex refined by the Non-uniform refinement in cryoSPARC, the resolution was assessed by the Gold Standard of FSC=0.143.

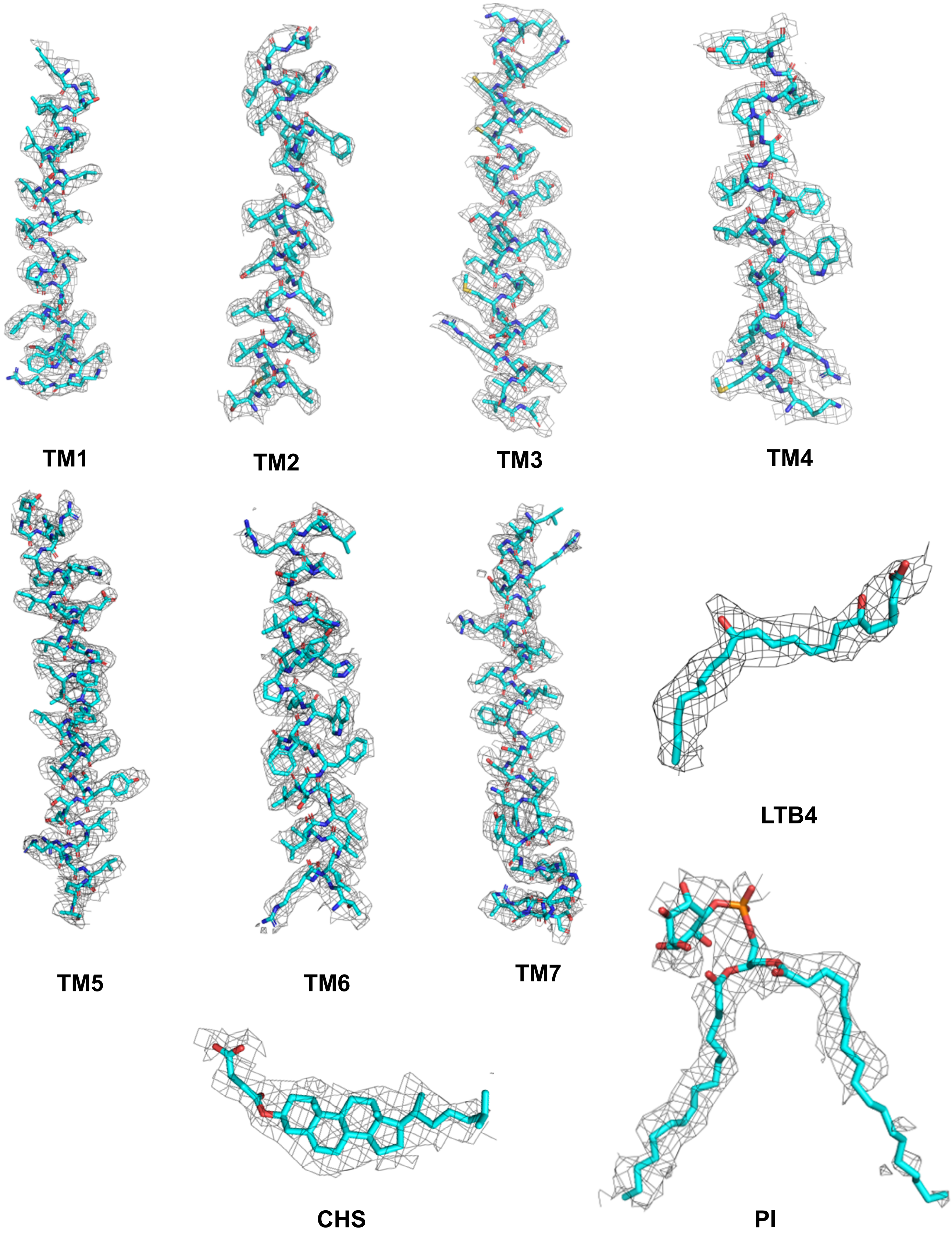

Supplementary Fig. 4. The cryo-EM density map of the representative regions of the BLT1/Gi complex. Contour level of 3.0-5.0.

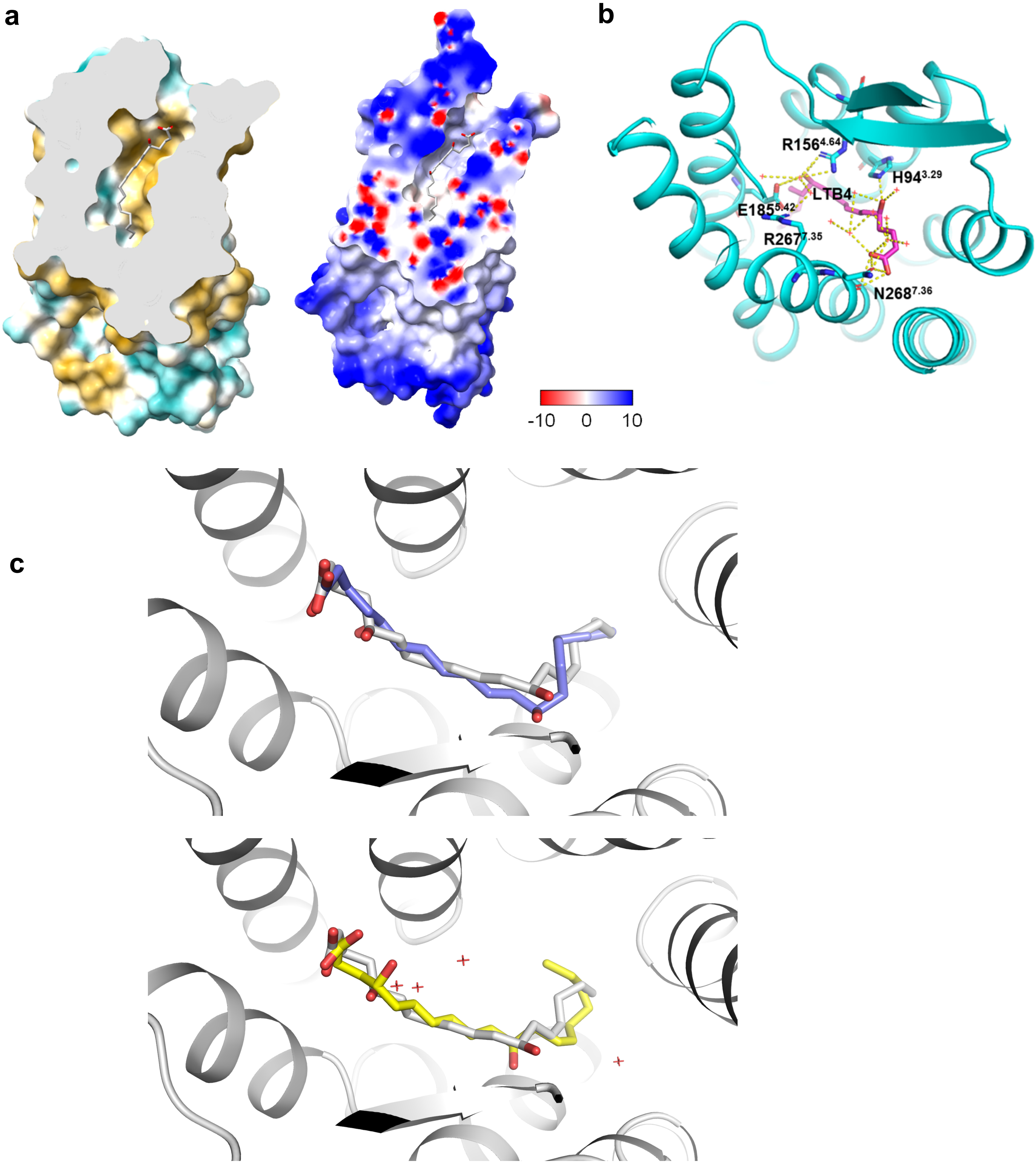

Supplementary Fig. 5. Additional data for the ligand binding pocket.
a, Cross section of a hydrophobicity (left panel) and electrostatic potential (right panel) analysis of the LTB4-bound BLT1. The light orange color indicates hydrophobicity and blue color indicates hydrophilicity. The blue color indicates positive charge and red color indicated negative charge. b, A snapshot of the MD simulation of LTB4-bound BLT1. The red points are waters in the ligand binding pocket. c, Docking analysis of the BLT1 ligand binding pocket. Upper panel, docking without water molecules, the white colored LTB4 is the original LTB4 in the cryo-EM structure, the blue colored LTB4 is the best docking result. Lower panel, docking in the presence of water molecules, the white colored LTB4 is the original LTB4 in the cryo-EM structure, the yellow colored LTB4 is the best docking result.

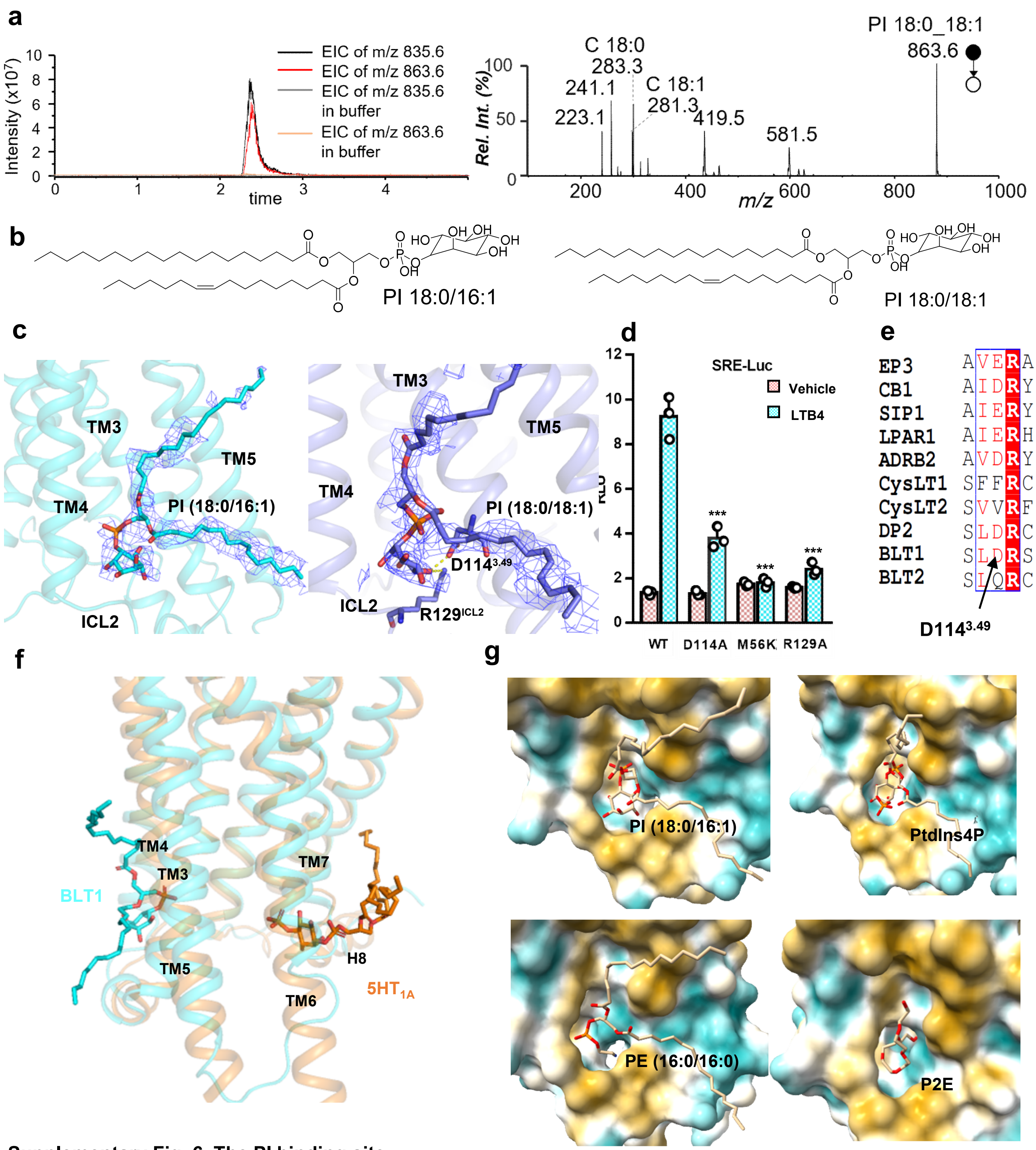

Supplementary Fig. 6. The PI binding site.

a, Additional MS analysis of the BLT1/G_i complex. b, The chemical structures of PI 18:0/16:1 and PI 18:0/18:1. c, The PI binding site overview. Left panel, model of PI 18:0/16:1; right panel, model of PI 18:0/18:1. The density map of PI (blue mesh) is set at contour level of 3.0. d, A SRE reporter assay of the PI binding site mutants. LTB4, 300 nM; data are presented as mean values ± SD; n =3 independent samples; n.s. no significant; *, $p < 0.05$; **, $p < 0.01$; ***, $p < 0.001$. The exact p value for D114A, M56K, R129A are: 0.0008, 0.0001, 0.0002, respectively. T-test, two tailed, sample equal variance. e, An alignment of the D114$^{3.49}$ among lipids receptor family. f, A comparison of the PI binding site of BLT1 (cyan) and the PtdINs4P binding site of $5HT_{1A}$ (gold). g, Model of PI 18:0/16:0, PtdIns4P and PE 17:0 in the PI binding site, the last one is the nonaethylene glycol (P2E) molecule in the pocket of MK-D-046-bound BLT1 (PDB:7k15).

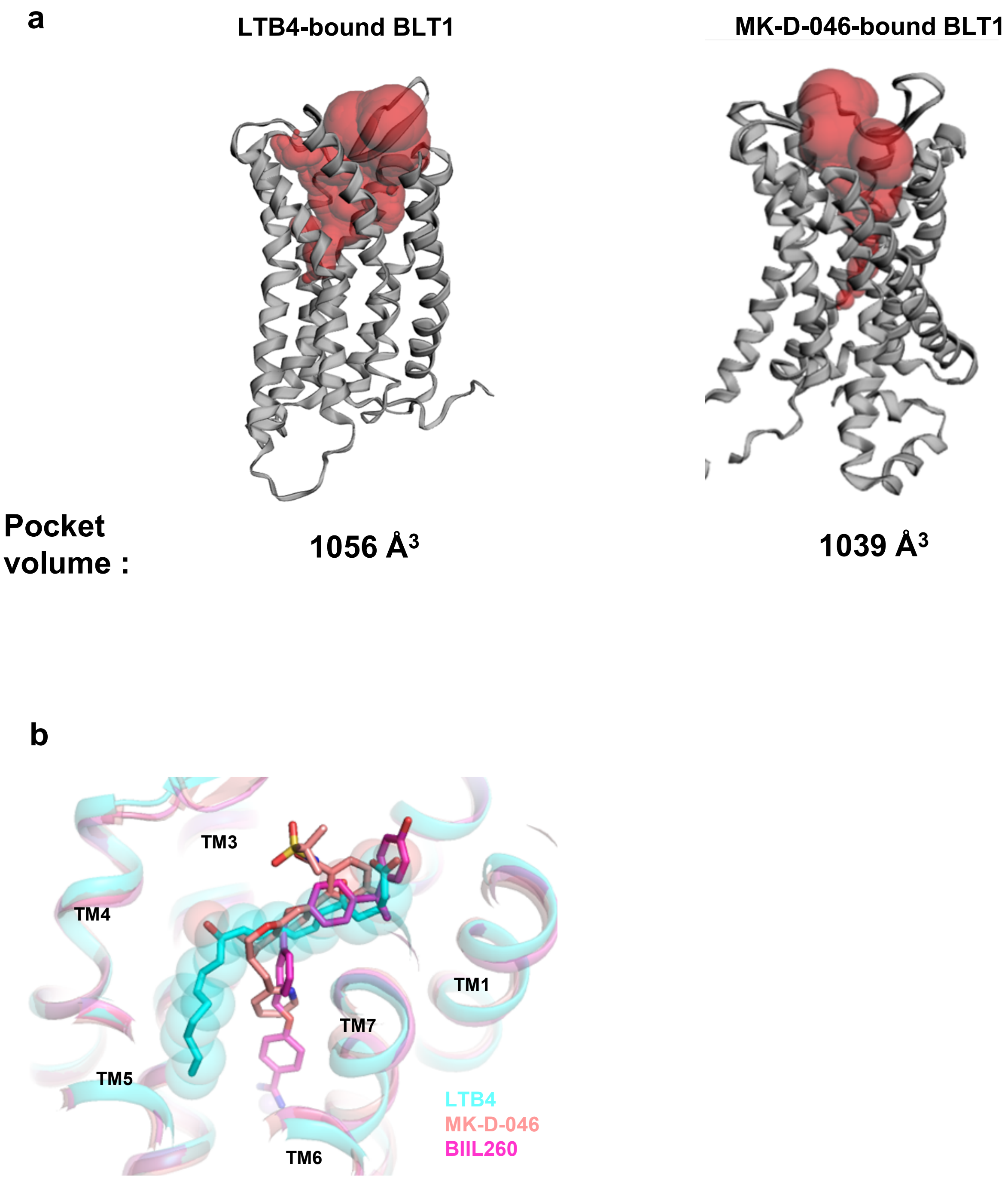

Supplementary Fig. 7. Additional information about the ligand binding pocket.
a, A comparison of the size of ligand binding pocket between the antagonist MK-D-046-bound BLT1 and the agonist LTB4-bound BLT1. The size of the ligand binding pocket was calculated by CASTp 3.0 server. b, The agonist LTB4 uses a different strategy to engage receptor. LTB4 binds to a tunnel close to TM3,4,5, while antagonist MK-D-046 (PDB:7k15) and BIIL260 (PDB:5x33) utilize the central tunnel of receptor.

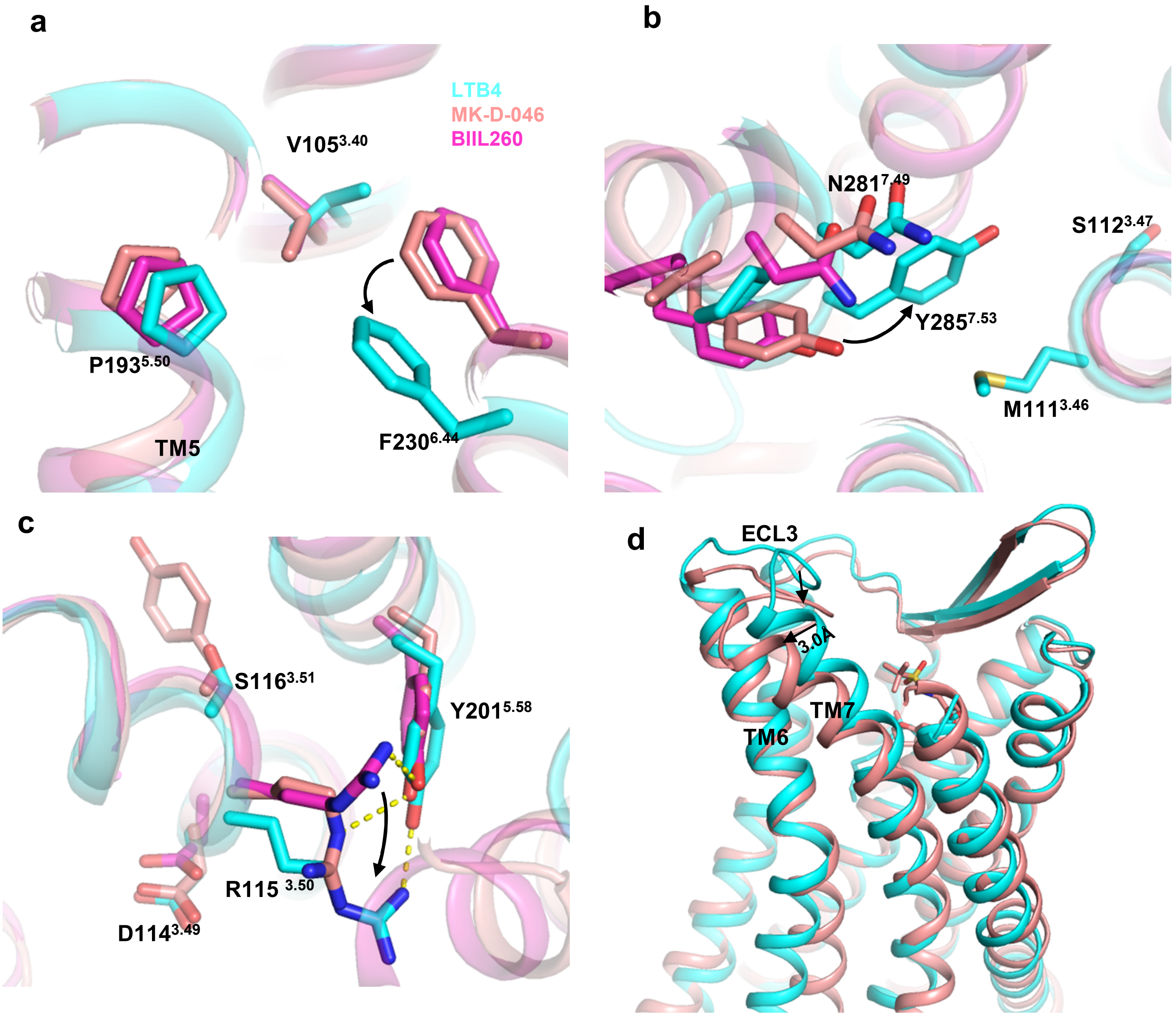

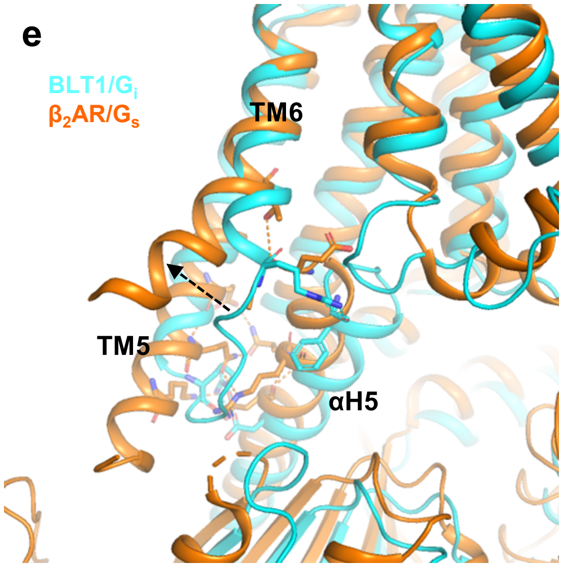

Supplementary Fig. 8. Conformational changes of the conserved motifs during receptor activation.
a, The conformational changes of the PIF motif upon receptor activation. b, The conformational changes of the NPxxY motif upon receptor activation. c, The conformational changes of the DRY motif upon receptor activation. d, The conformational difference of the extracellular side of TM7 and ECL3 between the agonist-bound (MK-D-046) and antagonist-bound (LTB4) receptor. e, A comparison of the BLT1/G_i complex with the β_2AR/G_s complex.

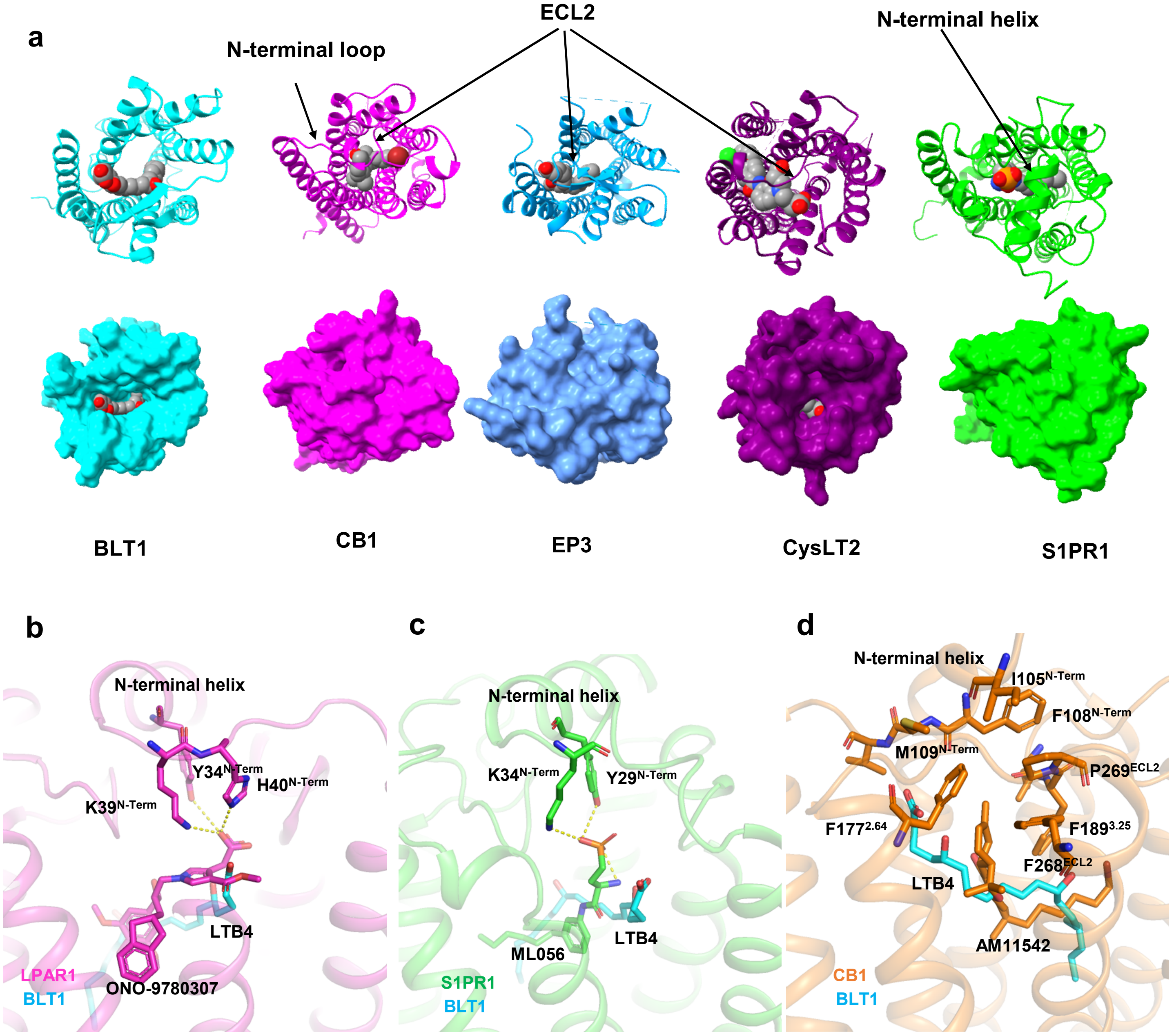

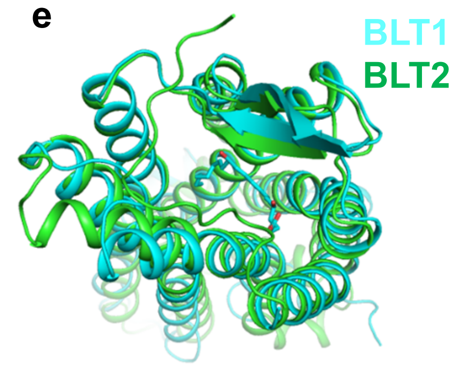

Supplementary Fig. 9. Top view of BLT1 with lipid receptors. a, A comparison of the upper ligand binding pocket among lipid receptor family. Upper panel, model of receptors; lower panel, surface of the receptor viewing from the top. CB1, PDB: 5xra; EP3, PDB:6ak3; CysTL2, PDB:6rz6; S1PR1, PDB:3v2y. b, The N-terminal helix of LPAR1 (PDB:4z34) forms hydrogen bond network with the ONO-9780307 compound. c, The N-terminal helix of S1PR1 (PDB:3v2y) forms hydrogen bond network with the ML056 compound. d, The N-terminal helix and the ECL2 of CB1 forms strong hydrophobic interaction with the AM11542 compound (PDB:5xra). In all cases, the BLT1 ligand LTB4 (cyan color) was superimposed with the corresponding lipid receptor, for better visual effect, the receptor part of BLT1 was hidden. e, A comparison of the upper ligand binding pocket between BLT1 and BLT2.

Supplementary Data Table 1| Cryo-EM data collection and refinement statistics

	BLT1/LTB4/Gαιβγ/scFv16<br>EMD-32018<br>7VKT
**Data collection and processing**	
Magnification	130,000
Voltage (kV)	300
Electron exposure (e^-/Å^2)	60
Defocus range (μm)	1.2-2.2
Pixel size (Å)	0.55
Symmetry imposed	C1
Initial particle image (no.)	2.9M
Final particle image (no.)	410k
Map resolution (Å)	2.9
FSC threshold	0.143
**Refinement**	
Initial model used (PDB code)	7k15, 6vms
Model Resolution (Å)	3.3
FSC threshold	0.143
Map sharpening *B* factor (Å^2)	-141.3
Model composition	
Non-hydrogen atoms	9094
Protein residues	1142
Ligands	5
B factor (Å^2)	
Protein	47.06
Ligand	67.48
R.m.s. deviations	
Bond length (Å)	0.003
Bond angles (°)	0.647
Validation	
MolProbity score	1.66
Clashscore	5.56
Poor rotamers (%)	0
Ramachandran plot	
Favored (%)	94.77
Allowed (%)	5.23
Disallowed	0
