# Supplementary material for: Structural basis of leukotriene B4 receptor 1 activation
Source: Nat Commun. 2022 Mar 3;13:1156. doi: 10.1038/s41467-022-28820-9 (PMC8894450; doi:10.1038/s41467-022-28820-9)
Supplement: Supplementary file 3 — Description of additional Supplementary File [file 41467_2022_28820_MOESM3_ESM.pdf]

## **Description of additional Supplementary data files**

Supplementary Movie 1. Movie track of LTB4-bound BLT1 receptor in MD simulation.
