# Supplementary material for: Structural basis of leukotriene B4 receptor 1 activation
Source: Nat Commun. 2022 Mar 3;13:1156. doi: 10.1038/s41467-022-28820-9 (PMC8894450; doi:10.1038/s41467-022-28820-9)
Supplement: Supplementary file 5 — Reporting Summary [file 41467_2022_28820_MOESM5_ESM.pdf]

nature research

Corresponding author(s): Yuanzheng He

Last updated by author(s): Jan 20, 2022

Reporting Summary

Nature Research wishes to improve the reproducibility of the work that we publish. This form provides structure for consistency and transparency in reporting. For further information on Nature Research policies, see our Editorial Policies and the Editorial Policy Checklist.

Statistics

For all statistical analyses, confirm that the following items are present in the figure legend, table legend, main text, or Methods section.

n/a	Confirmed	
☐	☒	The exact sample size (*n*) for each experimental group/condition, given as a discrete number and unit of measurement
☐	☒	A statement on whether measurements were taken from distinct samples or whether the same sample was measured repeatedly
☐	☒	The statistical test(s) used AND whether they are one- or two-sided<br>*Only common tests should be described solely by name; describe more complex techniques in the Methods section.*
☒	☐	A description of all covariates tested
☒	☐	A description of any assumptions or corrections, such as tests of normality and adjustment for multiple comparisons
☐	☒	A full description of the statistical parameters including central tendency (e.g. means) or other basic estimates (e.g. regression coefficient) AND variation (e.g. standard deviation) or associated estimates of uncertainty (e.g. confidence intervals)
☐	☒	For null hypothesis testing, the test statistic (e.g. *F*, *t*, *r*) with confidence intervals, effect sizes, degrees of freedom and *P* value noted<br>*Give P values as exact values whenever suitable.*
☒	☐	For Bayesian analysis, information on the choice of priors and Markov chain Monte Carlo settings
☒	☐	For hierarchical and complex designs, identification of the appropriate level for tests and full reporting of outcomes
☒	☐	Estimates of effect sizes (e.g. Cohen's *d*, Pearson's *r*), indicating how they were calculated

Our web collection on statistics for biologists contains articles on many of the points above.

Software and code

Policy information about availability of computer code

Data collection	EPU-v2.2.0
Data analysis	Relion 3.1, Rosetta 3.1, CryoSPARC 2.15, Pymol 2.3.2, Coot 0.89, Phenix 1.18.2, CASTp 3.0, MotionCor2, CTFFIND4.1, UCSF Chimera 1.15, crYOLO 1.7.4, MolProbity 4.1, Amber 20,CPPTRAJ v5.1.0

For manuscripts utilizing custom algorithms or software that are central to the research but not yet described in published literature, software must be made available to editors and reviewers. We strongly encourage code deposition in a community repository (e.g. GitHub). See the Nature Research guidelines for submitting code & software for further information.

Data

Policy information about availability of data

All manuscripts must include a data availability statement. This statement should provide the following information, where applicable:

- Accession codes, unique identifiers, or web links for publicly available datasets
- A list of figures that have associated raw data
- A description of any restrictions on data availability

All data produced or analyzed in this study are included in the main text or the supplementary materials. A reporting summary for this article is available as a Supplementary Information file. The cryo-EM density maps and atomic coordinates have been deposited in the Electron Microscopy Data Bank (EMDB) and Protein Data Bank (PDB) under accession numbers EMD-32018 and 7vkt for the LTB4-bound BLT1/Gi complex. All other published PDB codes cited in this paper are 7k15, 5x33, 5xra, 6ak3, 6rz6, 3v2y, 4z34 and 6vms. Source data are provided with this paper.

Field-specific reporting

Please select the one below that is the best fit for your research. If you are not sure, read the appropriate sections before making your selection.

[x] Life sciences   [ ] Behavioural & social sciences   [ ] Ecological, evolutionary & environmental sciences

For a reference copy of the document with all sections, see nature.com/documents/nr-reporting-summary-flat.pdf

Life sciences study design

All studies must disclose on these points even when the disclosure is negative.

Sample size	For all reporter functional assays, based on previous experience, three independent experiments (n=3-5) are carried out and sufficient.See figure legends for detail.
Data exclusions	No data were excluded
Replication	Key functional experiments were repeated at least two times independently,all attempts at replication were successful.
Randomization	Randomization is not required for this study as this is not a hypothesis driven study also based on the nature of structural biology.
Blinding	Blinding was not performed as this is not a hypothesis driven study and also because of the nature of structural biology.

Reporting for specific materials, systems and methods

We require information from authors about some types of materials, experimental systems and methods used in many studies. Here, indicate whether each material, system or method listed is relevant to your study. If you are not sure if a list item applies to your research, read the appropriate section before selecting a response.

Materials & experimental systems

n/a	Involved in the study
[x]	[ ] Antibodies
[ ]	[x] Eukaryotic cell lines
[x]	[ ] Palaeontology and archaeology
[x]	[ ] Animals and other organisms
[x]	[ ] Human research participants
[x]	[ ] Clinical data
[x]	[ ] Dual use research of concern

Methods

n/a	Involved in the study
[x]	[ ] ChIP-seq
[x]	[ ] Flow cytometry
[x]	[ ] MRI-based neuroimaging

Eukaryotic cell lines

Policy information about cell lines

Cell line source(s)	sf9 cell line, Invitorgen cat#11496-015; AD-293 cell line, Agilent # 240085
Authentication	No authentication required
Mycoplasma contamination	All cell lines tested are negative for mycoplasma contamination
Commonly misidentified lines (See ICLAC register)	No commonly misidentified cell lines were used
